# Postural Stability in Young Adults with Down Syndrome in Challenging Conditions

**DOI:** 10.1371/journal.pone.0094247

**Published:** 2014-04-11

**Authors:** Ewa Bieć, Joanna Zima, Dorota Wójtowicz, Bożena Wojciechowska-Maszkowska, Krzysztof Kręcisz, Michał Kuczyński

**Affiliations:** 1 Faculty of Physiotherapy, University School of Physical Education in Wroclaw, Wroclaw, Poland; 2 Department of Biomechanics, The Opole University of Technology, Opole, Poland; University of California, Merced, United States of America

## Abstract

To evaluate postural control and performance in subjects with Down syndrome (SwDS), we measured postural sway (COP) in quiet stance in four 20-second tests: with eyes open or closed and on hard or foam surface. Ten SwDS and eleven healthy subjects participated, aged 29.8 (4.8) and 28.4 (3.9), respectively. The time-series recorded with the sampling rate of 100 Hz were used to evaluate postural performance (COP amplitude and mean velocity) and strategies (COP frequency, fractal dimension and entropy). There were no intergroup differences in the amplitude except the stance on foam pad with eyes open when SwDS had larger sway. The COP velocity and frequency were larger in SwDS than controls in all trials on foam pad. During stances on the foam pad SwDS increased fractal dimension showing higher complexity of their equilibrium system, while controls decreased sample entropy exhibiting more conscious control of posture in comparison to the stances on hard support surface. This indicated that each group used entirely different adjustments of postural strategies to the somatosensory challenge. It is proposed that the inferior postural control of SwDS results mainly from insufficient experience in dealing with unpredictable postural stimuli and deficit in motor learning.

## Introduction

Individuals with Down syndrome exhibit several major motor disorders when compared to healthy persons. Slow movements, different gait patterns and an inability to respond rapidly to changes in the environment [1]–[3] are easily noticed even by casual observers. Much less apparent - although of fundamental importance in the overall motor development of these persons - is deteriorated postural stability [3]–[5]. Postural stability is a rate-limiting factor and a prerequisite for a great number of gross motor skills. Good stability supports more complicated movements, encourages us to explore new situations, and ultimately leads to optimal motor control, based on growing experience and skill in using a variety of motor synergies. In contrast, poor stability increases the risk of falling and puts constraints on physical activity, which results in very limited motor experience in subjects with Down syndrome (SwDS). Hence, improving postural control may be beneficial for these people for at least two reasons. Firstly, better stability usually leads to decreased sway and increased stability area, which may build self-confidence in SwDS, allowing them to interact with the environment more willingly, with less fear of falling. Secondly, with some of the internal constraints on physical activity presumably lifted, a broader repertoire of movements should occur. Apparently, habitual inactivity and inadequate postural control form a vicious circle in SwDS that must be broken to clear the way for their overall functional improvement.

To achieve this goal, we need to know the mechanisms of deteriorated postural control in SwDS. Once they are deciphered, the premises for adequate therapy will come to light; however, the existing literature seems vague on this subject. Poor postural stability was attempted to be explained through several cognitive and motor deficits found in these patients. Cognitive impairment may cause deteriorated information processing, slower decision making [2] and poorer ability to integrate multimodal sensory input. Other deficits include longer simple motor reaction times [6], excessive grip force [7], poorer capacity to adapt to changes in sensory information, delays in the onset of postural activity and loss of anticipatory postural control [1]. The SwDS also show different responses to novel tasks [8] than healthy subjects. Potentially, all of these deficits may affect postural control system; however, no study has provided a plausible explanation for the role of these deficits in deteriorated postural control in SwDS. It may signify the presence of another antecedent of the altered postural performance in SwDS, which is very likely related to specific postural strategies that have evolved in the process of reciprocal interactions during concurrent development of the postural and motor system.

In light of the aforementioned habitual inactivity and limited motor experience of SwDS, the possible reason for their balance deficit may be linked to an inadequately developed postural control system that does not adequately adjust its responses to the changing levels of postural task requirements. However, the traditional amplitude measures of postural performance are not well suited to assess this issue because of their descriptive character and low specificity. On the other hand, the nonlinear measures of sway dynamics, i.e., sway entropy and fractal dimension, have shown promise in detecting important differences between the groups investigated. The strength of the latter measures lies in their ability to detect between-group differences which may be attributed to different levels of automaticity and/or adaptability that are specifically adjusted according to the balance challenge. The richer the changes in challenge to balance we apply, the more complete the picture of the inter-group differences we might expect.

The main purpose of this study was to assess standing stability in SwDS in a series of trials with increasing postural challenge using traditional and nonlinear measures of the center-of-pressure (COP) time-series and to compare it with standing stability in healthy subjects. We hypothesized that the SwDS would exhibit deteriorated postural performance (higher values of the COP variability and mean velocity) and different postural strategies in comparison with the control subjects. Specifically, with regard to postural strategies, we hypothesized that SwDS would show inadequate changes in these strategies that would account for their insufficient preparation to the more demanding postural tasks. Further elucidation of these differences will help improve our understanding of the antecedents of falls, and provide a rational basis for designing rehabilitation programs for individuals with Down syndrome.

## Methods

### Subjects

The study was approved by the Senate Ethics Committee for Research at the University School of Physical Education in Wroclaw. All subjects and their guardians provided written informed consent to participate in the research.

Subjects from the Occupational Therapy Workshop participated in the research voluntary and were not disadvantaged in any other way by not participating in the study.

The study involved 10 people with Down syndrome who participated in the Occupational Therapy Workshop in Wroclaw and 11 doctoral students from our university. The basic characteristics of subjects, i.e., age, the type of Down syndrome and level of intellectual disability are presented in Table 1.

**Table 1:** Characteristics of the subjects with Down syndrome (SwDS, N = 10) and control group (CG, N = 11).

		SwDS	CG
**Sex**	Male	5	7
	Female	5	4
**Age**		29.8 (4.8)	28.4 (3.9)
**Type of Down syndrome**	Trisomy of chromosom 21	10	-
	Trisomy with translocation	-	-
	Mosaicism	-	-
**Intellectual disability**	Light	-	-
	Moderate	5	-
	Significant	5	-
	Deep	-	-

### Procedure

Postural stability was assessed in four 20-second trials of quiet standing on a hard or soft (a 50-kg/m^3^ foam pad placed on a platform) support surface with eyes open (EO) or closed (EC). The sequence of trials was fixed: EO followed by EC on a hard surface and then repeated on a soft surface with 1-minute breaks between consecutive trials. The subjects were asked to stand barefoot as motionlessly as possible with feet together and hands at their sides. In the EO trials the subjects were instructed to focus their gaze on a dot placed at eye level at a distance of 2 m. A practice run was allowed prior to the test to ensure that the subjects felt comfortable in the laboratory area. Each recording started 10 seconds after the subject was ready for testing to eliminate possible transients in the COP data.

### Data analysis

Data were recorded on a force plate (Kistler 9286 AA) at a sampling frequency of 100 Hz. The COP signal was calculated from the recorded ground reaction forces in the medial-lateral (ML) and anterior-posterior (AP) plane separately. Postural balance was evaluated by five parameters based on the COP signal: standard deviation (SD), mean speed (MV) [9], sample entropy (SE) [10], frequency (FR) and fractal dimension (FD). Measures of COP variability and mean speed determine performance, with lower values of these indices indicating better performance. The SE is the negative natural logarithm of an estimate of the conditional probability that a subseries (an epoch) of length m that matches pointwise within a specific tolerance r also matches at the next point [11]. High values of sample entropy are associated with a low probability of repeated template sequences in the data. In other words, the higher the sample entropy, the greater the irregularity of the time series. The increased values of sample entropy, which indicate larger irregularity of the COP, have been attributed to a reduced amount of attention invested in posture [12] and may be interpreted as an increase in the efficiency or ‘automaticity’ of postural control [13]. Input parameters for estimating the sample entropy were based on the median value of the relative error [11] resulting in the selection of pattern length of m = 3 and error tolerance of r = 0.02 as optimal parameters for both ML and AP planes (normalized to unit variance) of all subjects and tasks. A visual guide to optimal selection of the latter two parameters for sample entropy estimation may be found in Roerdink et al. [12] (see Appendix). The fractal dimension of the signal was calculated using custom-written software in Matlab (Higuchi's algorithm, see Appendix). Fractal dimension is an easily accessible measure that could be used for the study of COP complexity, and provides more information about posture control than traditional measures [14], [15]. The data were tested for normal distribution and homogeneity of variances. After log-transformation of the non-normally distributed data, all dependent variables were subjected to 2 groups (SwDS and CG)×2 surfaces (hard and compliant)×2 conditions (with eyes open and closed)×2 planes (AP and ML) ANOVA (Statistica 9.0) with repeated measures of the last three factors. Selected pairwise comparisons were explored using follow-up analyses (Tukey test). The level of significance was set at p<0.05.

## Results

The means and their standard deviation of the variables are shown in Table 2.

**Table 2:** The means and their standard deviation of the variables.

		Group	Platform		Foam pad	
			EO	EC	EO	EC
AP	SD (mm)	SwDS	3.8+1.2	5.2+0.7	11.3+1.2	16.6+2.1
		CG	3.7+1.9	5.2+1.9	9.3+3.5	14.3+6.8
	MV(mm/s)	SwDS	13.3+4.9	18.9+5.9	54.5+27.5	76.6+25.3
		CG	8.3+0.9	12.1+3.0	16.5+2.5	36.9+9.2
	FR (Hz)	SwDS	0.59+0.26	0.64+0.24	0.77+0.28	0.78+0.27
		CG	0.43+0.17	0.41+0.15	0.32+0.14	0.44+0.12
	SE (-)	SwDS	0.75+0.18	0.72+0.16	0.67+0.10	0.65+0.09
		CG	0.97+0.37	0.80+0.27	0.56+0.17	0.64+0.09
	FD (-)	SwDS	1.46+0.10	1.49+0.08	1.53+0.09	1.55+0.08
		CG	1.38+0.08	1.41+0.07	1.39+0.08	1.44+0.05
ML	SD (mm)	SwDS	4.1+1.5	4.7+2.2	10.2+3.12	12.3+2.9
		CG	3.4+1.1	5.9+2.2	5.5+0.8	13.2+2.4
	MV(mm/s)	SwDS	10.6+2.6	14.5+5.7	43.0+12.4	52.3+18.3
		CG	8.3+1.7	14.7+5.1	16.0+2.6	41.0+7.8
	FR (Hz)	SwDS	0.44+0.13	0.52+0.11	0.68+0.16	0.67+0.18
		CG	0.42+0.15	0.43+0.14	0.47+0.10	0.50+0.06
	SE (-)	SwDS	0.69+0.07	0.75+0.05	0.68+0.03	0.70+0.02
		CG	0.65+0.22	0.60+0.14	0.58+0.07	0.57+0.05
	FD (-)	SwDS	1.41+0.07	1.47+0.05	1.52+0.05	1.52+0.06
		CG	1.42+0.06	1.42+0.06	1.45+0.06	1.46+0.02

(SD- COP standard deviation, MV- COP mean velocity, FR- COP frequency, SE- COP sample entropy, FD- fractal dimension).

### COP standard deviation (SD)

**Figure 1:**
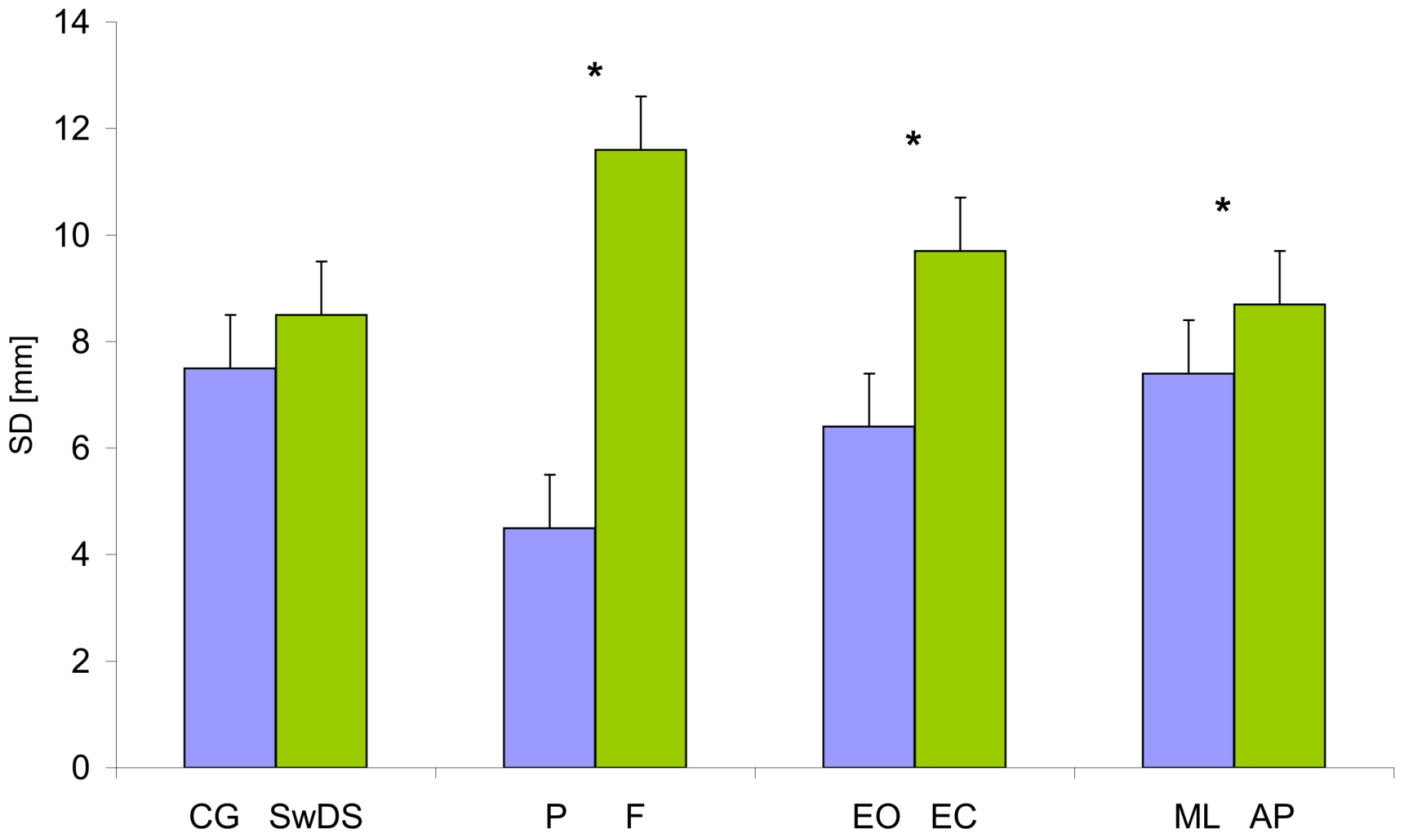
Mean values of the COP standard deviation (SD) collapsed over group, surface, eyes and plane. Vertical bars indicate the standard error. (CG- control group, SwDS- subjects with Down syndrome, P- platform, F- foam, EO- eyes open, CE- eyes closed, ML- medial-lateral plane, AP- anterior-posterior plane. Asterisks indicate significant differences (p<0.05) between conditions.)

**Figure 2:**
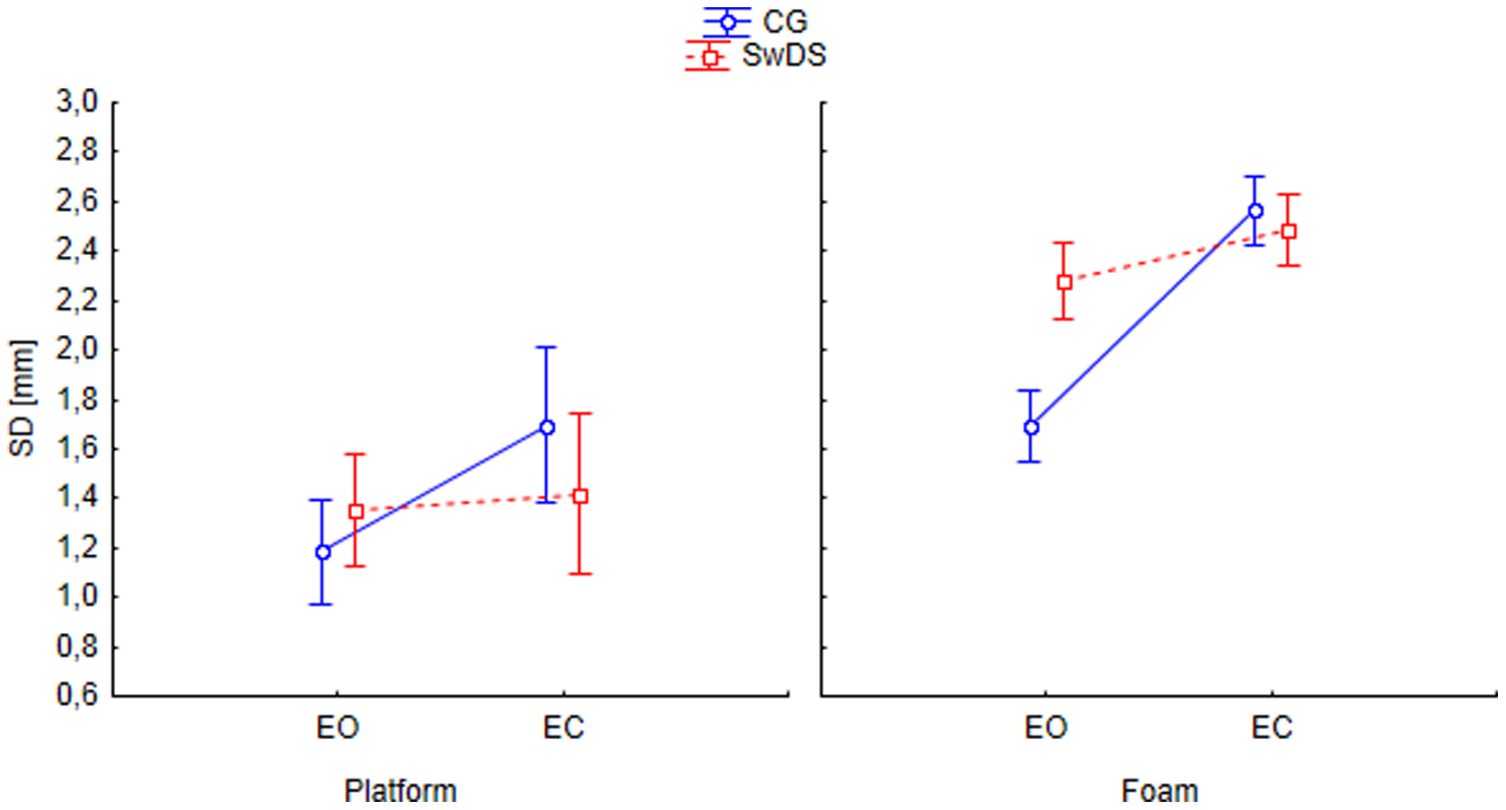
Group, eyes and surface interaction on standard deviation (SD) in medial-lateral plane. (CG- control group, SwDS- subjects with Down syndrome, EO- eyes open, EC- eyes closed.)

Surface, eyes and plane significantly affected COP standard deviation (Figure 1), F(1, 19) = 208.8, p<0.05; F(1, 19) = 52.4, p<0.05; F(1, 19) = 13.9, p<0.05, respectively as did the group×eyes F(1, 19) = 8.8, p<0.05 and the eyes×plane×group F(1, 19) = 12.6, p<0.05 interactions. The SD was larger on foam, with EC, and in the AP plane. Eyes closure equally affected both groups in the AP plane significantly increasing the COP amplitude. However, in the ML plane, the two groups behaved differently as depicted in Figure 2, in contrast to the controls, the SwDS displayed no differences between the EO and EC conditions. Additionally Figure 2 demonstrates an 85% higher COP SD in SwDS as compared to controls while standing on foam with EO (F(1, 19) = 14.0, p<0.001), being the only yet meaningful intergroup difference.

### COP mean speed (MV)

Group, surface, plane and eyes significantly affected COP mean speed, F(1, 19) = 24.8, p<0.05, F(1, 19) = 343.4, p<0.05, F(1, 19) = 7.9, p<0.05, F(1, 19) = 133.9, p<0.05, respectively as did surface×group F(1, 19) = 15.7, p<0.05, eyes×group F(1, 19) = 19.7, p<0.05, plane×group F(1, 19) = 19.1, p<0.05, group×surface×eyes F(1, 19) = 12.6, p<0.05 and group×eyes×plane F(1, 19) = 18.2, p<0.05 interaction. Mean speed was larger in SwDS than in CG, on foam than on hard surface, with eyes closed than with eyes open, and in AP than in ML plane. The group×surface interaction (F(1, 19) = 24.6, p<0.0001) showed that changing the support surface from hard to foam increased MV much more in the SwDS than in controls. The group×surface×plane interaction (F(1, 19) = 8.3, p<0.01) accounted for much larger contribution from AP than ML plane to this increase in MV on foam. Finally, the group×plane interaction (F(1, 19) = 17,3p<0.001) revealed that SwDS had much higher MV than controls in AP plane only.

### Fractal dimension (FD)

**Figure 3:**
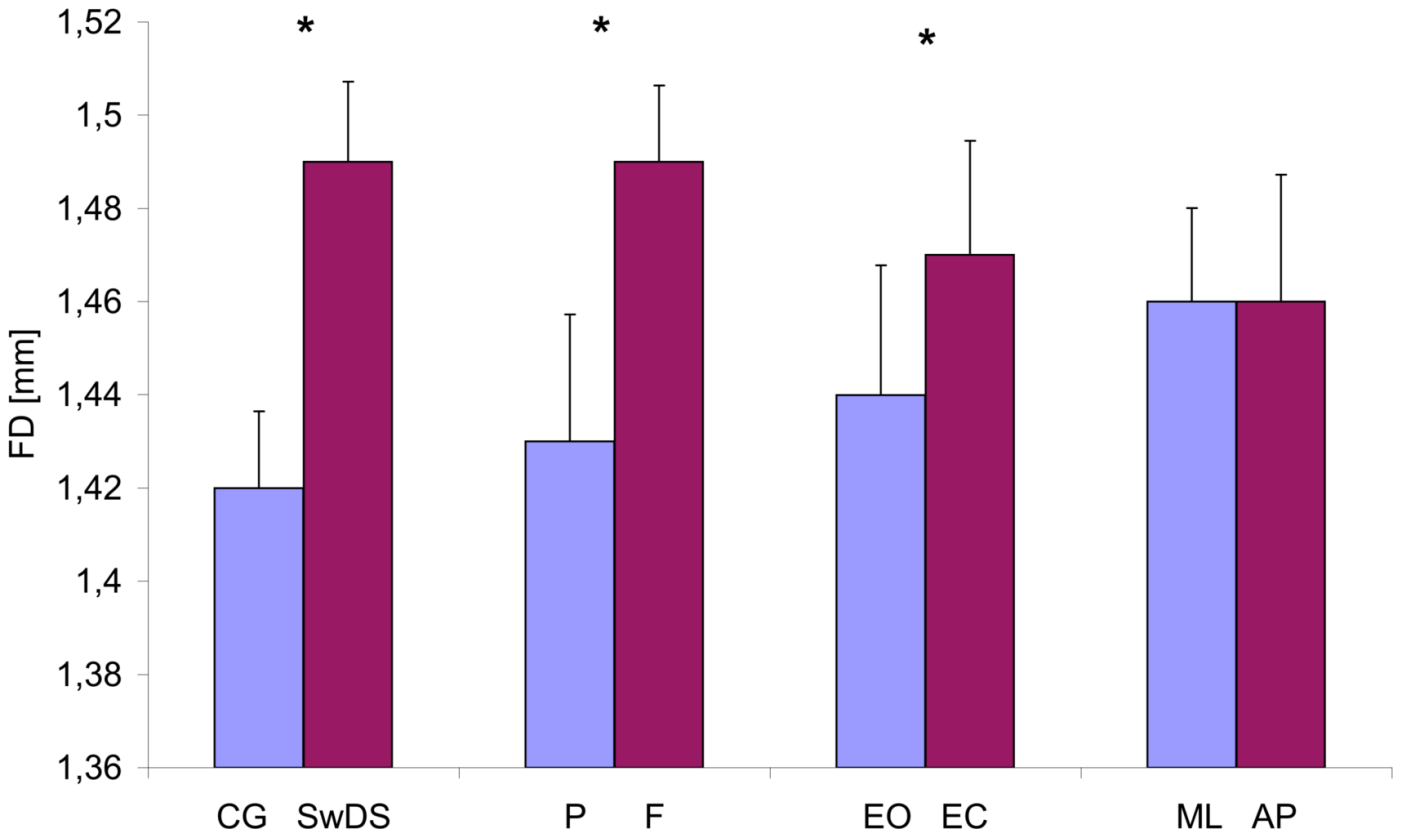
Mean values of the COP fractal dimension (FD) collapsed over group, surface, eyes and plane. Vertical bars indicate the standard error. (CG- control group, SwDS- subjects with Down syndrome, P- platform, F- foam, EO- eyes open, CE- eyes closed, ML- medial-lateral plane, AP- anterior-posterior plane. Asterisks indicate significant differences (p<0.05) between conditions.)

Group, surface and eyes significantly affected fractal dimension (Figure 3), F(1, 19) = 9.4, p<0.05, F(1, 19) = 23.3, p<0.05, F(1, 19) = 14.1, p<0.05, respectively as did group×plane F(1, 19) = 14.23, p<0.05, group×eyes×surface F(1, 19) = 6.6, p<0.05, and group×eyes×plane F(1, 19) = 8.0, p<0.05. The FD was higher in SwDS, on foam pad, and with EC. The group×plane interaction accounted for larger FD intergroup difference in the AP than in ML plane. The group×eyes×surface interaction indicated that the shift from a hard surface to foam pad had larger effect on SwDS in EO than EC without affecting the control group.

### COP sample entropy (SE)

**Figure 4:**
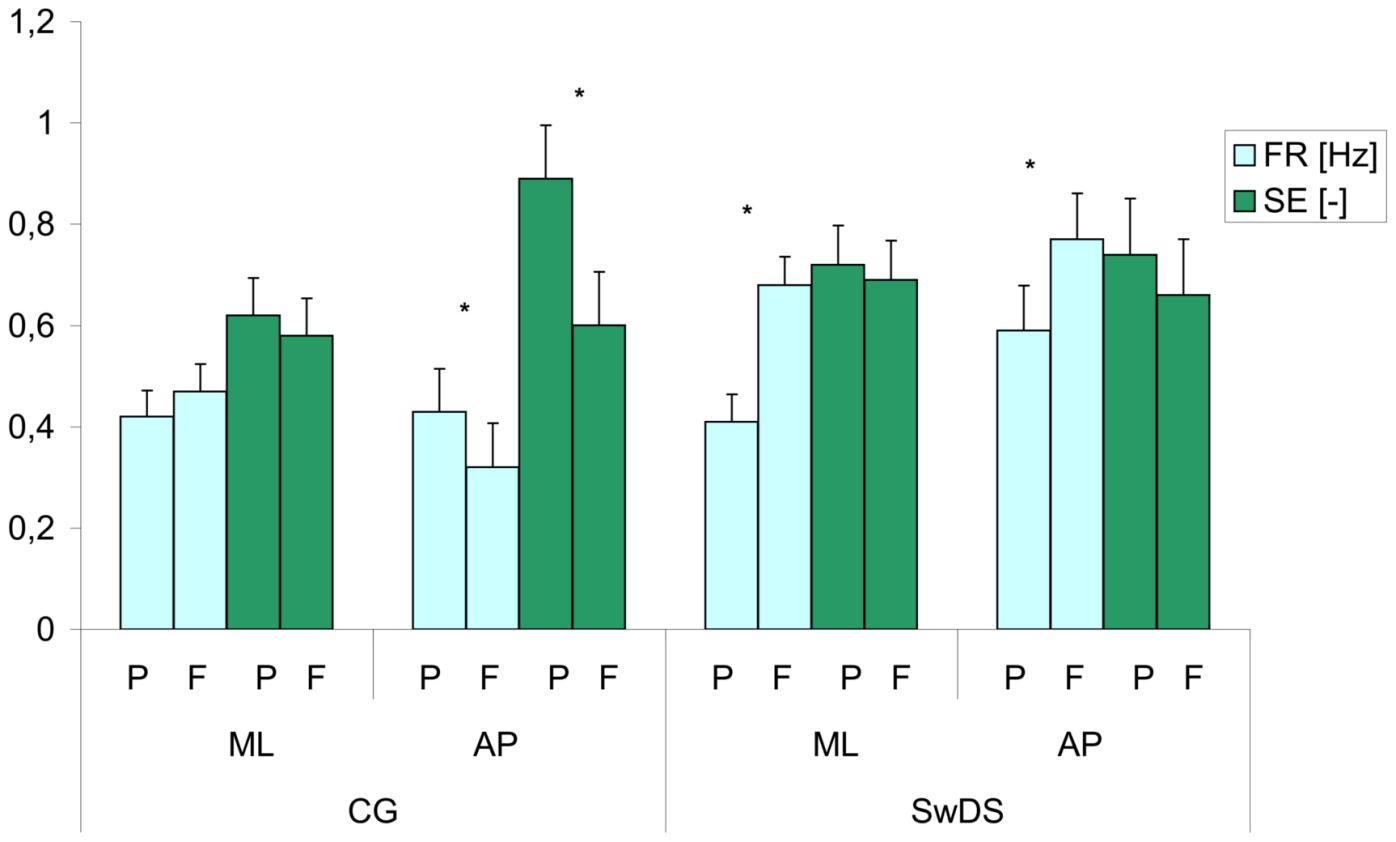
Mean values of the COP frequency (FR) and sample entropy (SE) in subjects with Down syndrome (SwDS) and control group (CG) in the medial-lateral (ML) and anterior-posterior (AP) planes while standing on the platform (P) and foam (F) with eyes open. Vertical bars indicate the standard error. Asterisks indicate significant differences (p<0.05) between stances on platform and foam.

Plane and surface significantly affected sample entropy, F(1, 19) = 8.4, p<0.05 and F(1, 19) = 11.1, p<0.05, respectively as did the group×plane interaction, F(1, 19) = 11.6, p<0.05. Higher values of sample entropy on the platform and in AP plane indicated more automatic postural control. The group×plane interaction showed that while the SwDS had the same level of automaticity in both planes, there was a difference in controls in favor of the AP plane. The group×surface×eyes interaction, F(1, 19) = 8.0, p<0.05, showed that the transition from hard to foam surface decreased SE in controls more in EO than in EC condition. In a similar way, the group×surface×plane interaction, F(1, 19) = 4.8, p<0.05 indicated that the transition from hard to foam surface significantly decreased SE in controls in AP plane only (Figure 4).

### COP frequency (FR)

**Figure 5:**
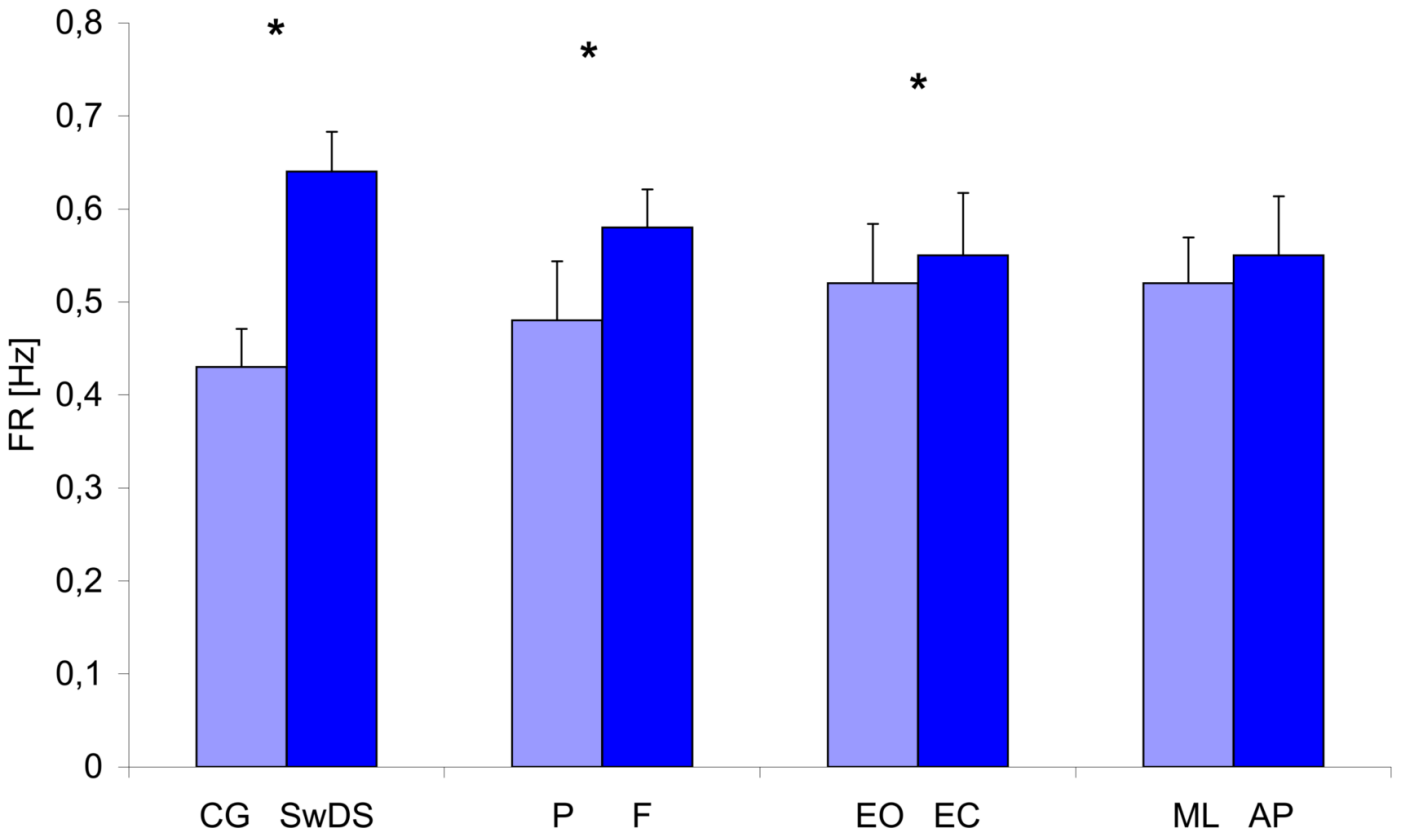
Mean values of the COP frequency (FR) collapsed over group, surface, eyes and plane. Vertical bars indicate the standard error. (CG- control group, SwDS- subjects with Down syndrome, P- platform, F- foam, EO- eyes open, CE- eyes closed, ML- medial-lateral plane, AP- anterior-posterior plane. Asterisks indicate significant differences (p<0.05) between conditions.)

Group, surface and eyes significantly affected COP frequency (Figure 5), F(1, 19) = 13,6, p<0.05, F(1, 19) = 14.0, p<0.05, F(1, 19) = 5.3, p<0.05, respectively as did the group×surface F(1, 19) = 8.1, p<0.05, the group×plane F(1, 19) = 14.5, p<0.05, and the group×eyes×surface F(1, 19) = 13.3 interactions. The COP frequency was higher in SwDS, on foam pad, and with EC. The group×surface interaction reflected the larger intergroup difference in FR on foam as compared to hard support surface which resulted solely from the FR increase in SwDS while the controls remained unaffected by the change of support surface. The group×plane interaction complemented the latter result indicating that AP plane only contributed to this difference with increased FR in SwDS and decreased FR in controls (Figure 4). The group×eyes×surface interaction indicated that the shift from hard surface to foam pad had larger effect on SwDS in EO than EC without affecting the control group.

## Discussion

The purpose of this study was to elucidate the mechanisms responsible for altered postural control in SwDS. We hypothesized that the SwDS would exhibit deteriorated postural performance (higher values of the COP variability and mean velocity) and different postural strategies in comparison with the control subjects. Although existing work on postural control in SwDS seemed to justify these hypotheses, they could be only partly verified.

Three results seem to be of particular interest. Firstly, postural performance based on the COP variability did not differ between groups in any tasks that might account either for similar postural behavior or effective compensatory strategies used by the SwDS. The only exception was much larger ML COP variability in SwDS during the EO stance on the foam pad. Secondly, the large increase in the COP MV and frequency caused by the foam pad in patients showed that this group might be particularly vulnerable to deteriorated or abundant somatosensory inputs. Thirdly, deteriorated somatosensory input had different effects on the sway measures in both groups. During stances on the foam pad, CON decreased sample entropy, exhibiting more conscious control of posture, while SwDS increased the fractal dimension, evidencing higher complexity of the equilibrium system in comparison to the stances on a hard support surface. This indicated that each group used entirely different adjustments in postural strategies to the somatosensory challenge. Fourth, the sway frequency, mean velocity, and fractal dimension were higher in SwDS than in the control group in the AP plane only. And last, the changes in sway frequency, sample entropy, and fractality, in response to the transition from hard surface to foam, affected only the SwDS in the eyes open condition. This accounts for particular vulnerability of these patients to the incongruent sensory inputs.

The COP variability, represented by standard deviation of postural sway, is a widely accepted measure of postural performance. In this study we did not find differences in COP variability between SwDS and the control group in any trial except the EO stance on the foam pad that revealed greater ML sway in the SwDS. This evidence indicated very similar postural performance in both groups. Our SwDS somehow managed to equal the control group. We must admit that several authors reported poorer performance in subjects with DS [4], [16], [17], but our results still concur with a recent study by Cabeza-Ruiz et al. [18] who compared COP variability in 27 individuals with DS and 27 healthy adults, and found no differences between both groups in EO or EC stances on a hard support surface. Our study extends these results to stances on a compliant support surface and collectively shows similar patterns of postural performance and similar changes in this performance resulting from altered visual and/or somatosensory inputs. At this point, based on the COP variability only, we would advocate the notion of quantitative rather than qualitative differences in postural control between SwDS and healthy adults [4], [17]. This notion argues that postural control mechanisms basically have similar principles in both groups, with a comparable weighting of sensory inputs [4].

The COP amplitude measures provide summary characteristics of the postural control system which have been often used for the general descriptive assessment and classification of postural performance in terms of better or worse. The main disadvantage of these measures is their inability to provide relevant interpretable information about the operation of the physiological control system [16]. Here, based on the COP variability, we found that the postural performance of both groups was about the same. Does this imply that the means undertaken by the CNS to produce the observed performance in both groups were also similar? The evidence gathered by the majority of researchers indicates the contrary, with a much higher COP mean velocity in SwDS than in control groups, reported even in simple standing trials [4], [17], [18]. Unfortunately, in too many studies the COP mean velocity is regarded as one more measure of postural performance that, if no more dependent variables are available, inevitably cuts out further inferences. Only Vuillerme et al. [4] interpreted higher mean velocity in teenagers with DS as more effort invested in balance control and correctly suggested a link between this effort and the increased sway activity which might be explained by increased cocontraction of antigravity muscles [2], [19]. In fact, a coactivation pattern in SwDS that involves the simultaneous increase in activity of agonist and antagonist muscle has been reported by several authors [6], [20], [21]. In addition, studies on postural control in healthy subjects have shown that this coactivation pattern, induced by specific instructions or experimental setup, has led to higher postural stiffness with accompanying higher frequency of sway [22]. Furthermore, some authors have actually compared sway frequency or stiffness between SwDS and CON. Webber et al. [16] found a positive relationship between sway velocity and postural stiffness, and higher values of these parameters in SwDS. Larger COP frequency with the accompanying larger COP mean velocity was reported by Rigoldi et al. [5] and Cabeza-Ruiz et al. [18].

The former scheme would imply the CNS's ability to perceive and evaluate the threat to stability imposed by alteration in the somatosensory input and to decide which level of postural stiffness and frequency should be selected and used. This gradual adjustment of stiffness and frequency to increasing postural challenge has been observed in healthy individuals [23] and explained as a need to perform faster postural corrections and/or exploratory function. Accordingly, within this scheme, postural control in SwDS would still be qualitatively similar to that of healthy subjects, with inferior stability on the foam pad attributed to very limited practice in this unstable environment. If this is true, it would be good news for SwDS, i.e., an extensive training that involves quick unpredictable changes in somatosensory input might improve stability. Our cautious optimism follows at least three observations in SwDS: (1) the lack of increased frequency on a hard support surface even in trials with EC in this study; (2) the decrease in postural stiffness over consecutive trials [16]; and (3) the improvement in performance and gradual substitution of cocontraction with coactivation patterns of muscle recruitment following intensive practice [6], [20]. This belief is further corroborated by Latash et al. [24] who pointed out that “persons with Down syndrome have all the machinery, both muscular and neural, to perform movements with characteristics like those seen in persons without Down syndrome.”

On the other hand, the “safety catch” scheme suggests that the CNS selects cocontraction as the best remedy for any disturbance regardless of its direction and (probably) magnitude. This protective strategy is often used by novices in the early stages of acquiring a new motor skill [2] which again implies that postural control in SwDS may not differ much from that of healthy persons. Still, healthy individuals use this simple strategy as a transient protection, which, following the progress in motor learning, is quickly replaced by a more effective reciprocal strategy [2]. Thus, cocontraction seems a common necessary postural strategy which is used by the CNS in more challenging situations. For one thing it manifests a sort of helplessness in establishing the optimal coordination pattern, and for another it provides the room and temporal space to endure and may facilitate adaptation.

In the present experiment, the CG did not need any “safety catch,” as can be judged from the lack of increased sway frequency on the foam pad. In contrast, the same trials turned out to be quite challenging for the SwDS, who relied on cocontraction that resulted in increased postural stiffness and frequency of sway. This apparent difference between the two groups may be tentatively explained by a physiological trigger that caused the onset of cocontraction in SwDS only. This was in no way surprising, as persons with poor postural control should detect threats to stability earlier than healthy individuals. In other words, the CNS of the SwDS would shift postural strategy from normal to stiff in response to lower values of sway amplitude or velocity and/or lower perceived distance to stability limits. We believe that these lower thresholds in SwDS are mainly the results of habitual physical inactivity, unwillingness to explore alternative strategies, and the basic goal of remaining safe [24]. When these factors are considered together, the SwDS have very limited experience with proper assessment of atypical stimuli, let alone quick and efficient decision making based on these stimuli. Thus, the “safety catch” strategy which is put into execution before the actual danger to posture arises seems to be an easy, comfortable and safe response to postural threats to SwDS.

To summarize, the traditional parameters of the COP indicate that postural stability (sway amplitude) during well-trained stances on a hard surface is equal in both groups. Yet, to achieve a similar level of performance in a novel environmental condition (standing on a foam pad) the SwDS need to use higher postural stiffness reflected by increased sway frequency and mean velocity. Such means of stabilizing the body are plainly excessive in comparison to control groups and resemble those used by healthy subjects at the early stage of motor development, as well as by subjects who are acquiring new motor skills or adaptations. A common characteristic of this initial stage of motor learning is the involvement of a broad spectrum of available sensory resources in order to select, fine-tune and integrate the optimal set of inputs later used to trigger efficient postural synergies. This implies that the postural control system at this transient developmental or learning stage is particularly plastic, multimodal, adaptive and complex. However, it also suggests that the SwDS may have some delay in motor learning abilities or that these abilities are vulnerable to environmental challenges. The nonlinear stabilographic parameters computed in this study seem to support the above interpretation and shed more light on the possible mechanisms involved in the postural control deficit of the SwDS. Higher levels of complexity and adaptivity have been associated with higher values of the COP fractality [15], [25], [26]. This fits well with our data which shows an increase in FD in all conditions on the foam pad for SwDS only, indicating that it was the patients but not the control group who treated the more demanding stances as those yet to be learned. In the control group the increased FD was observed only in more challenging conditions, such as in the ML plane with EC, where their postural automaticity was lowest. On the other hand, the decrease in the COP entropy, which was caused in the control group by foam pad stances, occurred only in conditions with the highest level of automaticity (AP plane with EO or EC).

These findings, which manifest an entirely different postural reaction demonstrated by different changes in SE and FD in both groups to the decreased reliability of somatosensory input, provide insight into sensory mechanisms of postural control. Standing on a compliant surface results in a significant challenge to postural balance and is often used to investigate the relative contributions from visual, somatosensory and vestibular systems [27], most frequently to test vestibular patients [28]. However, the additional role of this challenge in the present study was to tax the sensory response of our subjects in terms of automaticity and adaptability. Decreased entropy on a foam pad in healthy individuals concurs with the reports of other authors [29], [30] and may account for the selection of an optimal level of automaticity to the difficulty of the postural task. In fact, Strang et al. [30] suggested that the broad, continuous and deliberate sway reflects the most appropriate postural strategy available in this setting. Such a strategy was evident in our control group, who invested more attention in postural control by trying to adjust their postural behavior to the constraints imposed by a compliant surface. This may reflect their willingness to explore the new environment and possibly restructure the respective postural synergies based on perceived feedback. A similar conscious action was missing in the SwDS who, as shown by the increased COP FD, turned to higher complexity and adaptability as a means to deal with the somatosensory challenge. However, the increased complexity in biological signals has been shown to correlate positively with the number of neurons involved [31], suggesting that the SwDS probably used abundant means to cope with the experimentally impaired somatosensory input, which might obstruct their ability to identify relevant features of the task performed. Higher values of the FD have been argued to be associated with the tendency for instability or the use of less stable control strategies [14], [15]. Our results extend this proposition by suggesting a tentative yet plausible mechanism which may cause the instability as reflected by the increased FD. Excessive sensory inputs were sending a stream of information that was probably poorly organized and coordinated, and thus could lead to sensory conflict, which instead of being helpful in motor learning, might even endanger a stable stance. Along similar lines, Cimolin et al. [26] have interpreted the higher complexity of the COP in subjects with Prader-Willi syndrome as the inability to synergically modulate the three sensory systems involved in maintaining posture. This notion is further corroborated by the lack of effect of vision on the ML sway variability in SwDS, which accounts for the deficit in sensory integration.

We believe that the relationship between changes in sway performance and strategies in our SwDS, who were exposed to a series of increasingly challenging conditions, indicates mainly central origin or their postural deficit. However, it is known that the peripheral proprioceptive system may be altered in these patients due to hypotonia and ligament laxity [32], [33]. This may lead to the reduced proprioceptive acuity and to impaired reflex function. Reduced proprioceptive acuity is an important factor contributing to postural control feedback while the abnormalities of musculoskeletal reflex function may account for particularly large intergroup differences in the stances on foam. It is possible that these impairments may be to some extent compensated by the proper selection of postural strategies. Therefore, the change in postural strategies found in this study may partly underlie a dysfunction of the peripheral proprioceptive system.

Our results regarding the possible differences in postural control between the ML and AP plane seem equivocal. The only intergroup difference in postural performance (indexed by the sway standard deviation) occurred in the ML plane (with eyes open on foam pad), with SwDS performing worse than the controls. On the other hand, SwDS had higher values of sway frequency, mean velocity and fractal dimension than control subjects in the AP plane only, i.e. in the plane where no differences in performance were found. It indicates that the SwDS may have implemented an efficient postural strategy that allowed them to improve their AP stability. However, the adopted changes in the latter sway measures seem to be suboptimal leading to a question whether they should or should not be subjected to therapeutic interventions.

Collectively, it seems plausible that a major difference in postural control between patients with DS and healthy subjects is reflected in novel or relatively unpracticed situations which require either learning or selecting and reweighing the previously stored motor synergies. What can be learned from these findings to make the therapy for SwDS more efficient? It has been suggested as necessary to provide these patients with stimulating and diversified practice conditions in which they may learn how to perform motor tasks optimally. While we generally agree with the significance of such an environment, the present results postulate focusing increased attention on the early stages of acquiring control over novel postural tasks. We believe it is crucial to double-check the patients' understanding of instructions and to provide them with augmented feedback regarding the actual performance or knowledge of results. Furthermore, the process of selection of postural synergies should be actively supported by assessment of how the individual sensory inputs are perceived and how these inputs help in proper decision making. This is related to gaining information about many aspects of movement through inherent feedback compared to the reference of correctness and this process may be inadequately developed in persons with DS. However, our present knowledge on possible relationships between progress in postural learning and nonlinear measures of postural sway is only emerging, and, to avoid unnecessary speculation, further research in this area is warranted.

## Supporting Information

Appendix S1 **In this appendix we explain choice of the parameters** ***m*** **and** ***r*** **for computation of sample entropy and the parameter** ***k_max_*** **for computation of Higuchi fractal dimension.**
(DOC)
