# Supplementary material for: Postural Stability in Young Adults with Down Syndrome in Challenging Conditions
Source: PLoS One. 2014 Apr 11;9(4):e94247. doi: 10.1371/journal.pone.0094247 (PMC3984118; doi:10.1371/journal.pone.0094247)
Supplement: Appendix S1 — In this appendix we explain choice of the parameters m and r for computation of sample entropy and the parameter kmax for computation of Higuchi fractal dimension. (DOC) [file pone.0094247.s001.doc]

# Choice of the parameters m and r for computation of sample entropy

To estimate the sample entropy (SE), the COP time series were first normalized to obtain samples with zero-mean and unit standard deviation. Sample entropy is quantiﬁed as the negative natural logarithm of the conditional probability (CP = A/B) that a dataset of length N, having repeated itself within a tolerance r for m points, will also repeat itself for m + 1 points, without allowing self-matches. B represents the total number of matches of length m while A represents the subset of B that also matches for m + 1. Sample entropy thus follows from -log (A/B) [S1].

The computation of sample entropy requires the selection of m and r. Ramdani et al. [S2] proposed an empirical approach developed in two steps:

1. SE is computed for different values of m (m = 1, 2, 3 and 4) and r (r = 0.01 to 1) over the whole set of data and by separating the AP and ML planes. We selected m = 3 since we observed pseudo-convergence for m>3 for almost all r values in both AP and ML planes (see Figure S1, upper panels).
2. The second step is to use the maximal relative error of the SE and CP estimation proposed by Lake et al. [S3]. This metric simultaneously penalizes CP near 0 and near 1. The criterion was set to be no higher than .05, implying that the 95% conﬁdence interval of the sample entropy estimate is maximally 10% of its value. This step allows us to determine the value of r (for m = 3) which is associated to the lowest median of maximum relative errors over our data set. In both planes, AP and ML, for r = 0.02, the minimum values was reached (see Figure S1, lower panels).

Hence, the two optimal input parameters used for computing SE were m = 3 and r = 0.02 for both ML and AP planes. We performed these calculations using software from PhysioNet [S4].

Figure S1. Optimal selection of template length m and tolerance range r for sample entropy estimation. The upper panels depict corresponding sample entropy values for various combinations of m and r, showing convergence for m > 3. The lower panels show selected r based on minimum of maximum relative error in CP and sample entropy estimates.

# Choice of the parameter k_max_ for computation of Higuchi fractal dimension

Higuchi [S5] proposed the method to estimate fractal dimension (FD) of a fractal curve that may be applied directly to the time series. Higuchi’s algorithm is based on the measures of the mean length of the curve L(k) by using a segment of ksamples as a unit of measure. One takes the series representing the signal under consideration (samples taken at a regular interval):

a(1), a(2), a(3),..., a(N)

where a(i) is the signal amplitude at the i-th discrete point (moment of time) (i =1,...,N) and N is the total number of points. From this one then constructs k new time series,

a(m,k): a(m,k): a(m), a(m+k), a(m+2k), ... , a(m+int[(N-m)/k] * k) (m=1,2,...,k)

where int[...] denotes the greatest integer not exceeding the number in the square brackets; m and k are integers indicating, respectively, the initial time and the time interval.

The length, L m (k), of each curve a(m,k) is then calculated as:

L_m_(k) = {[S|a(m+i*k)-a(m+(i-1)|]*(N-1)/[(int[(N-m)/k)])*k]}/k

i=1,int [(N-m) / k]

where N is the total number of samples and (N-1)/[(int[(N-m)/k]) * k] is a normalization factor.

The length of the curve for the time interval k, L(k), is calculated as the mean of the k values L_m_(k) for m=1,2,...k:

L(k) = (SLm(k))/k m=1,k

The procedure is repeated for several k=1,2,...,k_max_ . If the L(k) value is proportional to k^-FD^ , the curve is fractal-like, with the fractal dimension FD. FD is easily evaluated as the angular coefficient of the linear regression of the graph ln(L(k)) versus ln(1/k) [S6].

The choice of k_max_ has some influence on the results and should be selected appropriately. Determining k_max_ was by a process of examining the data and plotting the fractal dimension over a range of k_max_; the point at which the fractal dimension plateaus was considered a saturation point beyond which no benefit could be gained from further calculations. However, for our data no clear saturation were found, so we chosed maximum possible value N/4 = 500 for both AP and ML planes [S7].

# References

1. Roerdink M, Hlavackova P, Vuillerme N (2010) Center-of-pressure regularity as a marker for attentional investment in postural control: A comparison between sitting and standing postures. Human movement science 30: 203- 212.
2. Ramdani S, Seigle B, Lagarde J, Bouchara F, Bernard PL (2009) On the use of sample entropy to analyze human postural sway data. Medical Engineering & Physics 31: 1023–1031.
3. Lake DE, Richman JS, Griffin MP, Moorman JR (2002) Sample entropy analysis of neonatal heart rate variability. American Journal of Physiology: Regulatory, Integrative and Comparative Physiology 283: R798- R797.
4. Goldberger AL, Amaral LA, Glass L, Hausdorff JM, Ivanov P, Mark RG, Mietus JE, Moody GB, Peng CK, Stanley HE (2000) PhysioBank, PhysioToolkit, and PhysioNet: components of a new research resource for complex physiologic signals. Circulation 101: e215–e220.
5. Higuchi T (1998) Approach to an irregular time series on the basis of the fractal theory. Physica D 31: 277-283.
6. Błaszczyk JW, Klonowski W (2001) Postural stability and fractal dynamics. Acta Neurobiologiae Experimentalis 61: 105-112.
7. Doyle TLA, Dugan E,. Humphries B, Newton RU (2004) Discriminating between elderly and young using a fractal dimension analysis of center of pressure. International Journal of Medical Science 1(1): 11-20.
